# Prazosin-Conjugated Matrices Based on Biodegradable Polymers and α-Amino Acids—Synthesis, Characterization, and *in Vitro* Release Study

**DOI:** 10.3390/molecules200814533

**Published:** 2015-08-12

**Authors:** Ewa Oledzka, Anna Sawicka, Marcin Sobczak, Grzegorz Nalecz-Jawecki, Agata Skrzypczak, Waclaw Kolodziejski

**Affiliations:** 1 Department of Inorganic and Analytical Chemistry, Faculty of Pharmacy with the Laboratory Medicine Division, Medical University of Warsaw, Banacha 1, Warsaw 02-097, Poland; E-Mails: sawicka.anna.maria@gmail.com (A.S.); marcin.sobczak@wp.pl (M.S.); waclaw.kolodziejski@wum.edu.pl (W.K.); 2 Department of Environmental Health Science, Faculty of Pharmacy with the Laboratory Medicine Division, Medical University of Warsaw, Banacha 1, Warsaw 02-097, Poland; E-Mails: gnalecz@wum.edu.pl (G.N.-J.); agata.skrzypczak@wum.edu.pl (A.S.)

**Keywords:** controlled release/delivery, macromolecular drug delivery, biodegradable polymers, polymeric drug delivery system, prazosin, cytotoxicity

## Abstract

Novel and promising macromolecular conjugates of the α1-adrenergic blocker prazosin were directly synthesized by covalent incorporation of the drug to matrices composed of biodegradable polymers and α-amino acids for the development of a polymeric implantable drug delivery carrier. The cyto- and genotoxicity of the synthesized matrices were evaluated using a bacterial luminescence test, protozoan assay, and *Salmonella typhimurium* TA1535. A new urethane bond was formed between the hydroxyl end-groups of the synthesized polymer matrices and an amine group of prazosin, using 1,1′-carbonyldiimidazole (CDI) as a coupling agent. The structure of the polymeric conjugates was characterized by various spectroscopy techniques. A study of hydrogen nuclear magnetic resonance (^1^H-NMR) and differential scanning calorimetry (DSC) thermodiagrams indicated that the presence of prazosin pendant groups in the macromolecule structures increased the polymer’s rigidity alongside increasing glass transition temperature. It has been found that the kinetic release of prazosin from the obtained macromolecular conjugates, tested *in vitro* under different conditions, is strongly dependent on the physicochemical properties of polymeric matrices. Furthermore, the presence of a urethane bond in the macromolecular conjugates allowed for obtaining a relatively controlled release profile of the drug. The obtained results confirm that the pharmacokinetics of prazosin might be improved through the synthesis of polymeric conjugates containing biomedical polymers and α-amino acids in the macromolecule.

## 1. Introduction

Prazosin belongs to a family of selective antagonists of α1-adrenergic receptors [1]. It was the first α1-blocker introduced for the treatment of hypertension, acting in the expansion of the smooth muscle of small blood vessels and thereby lowering blood pressure [2]. Prazosin action involves vasodilation by the selective blocking of α1-adrenergic receptors located in the walls of blood vessels, direct inhibition of phosphodiesterase, as well as increasing cyclic adenosine monophosphate (cAMP) and cyclic guanosine monophosphate (cGMP) amounts. The blocking of adrenergic receptor α1 inhibits vasoconstriction induced by endogenous catecholamines. Prazosin also reduces cardiac preload and thus leads to a small increase in cardiac output and heart rate. It is also believed that prazosin works within the central nervous system, where it inhibits the sympathetic system and modulates the activity of baroreceptors [2]. The effect of prazosin on lipid metabolism is also important, manifested by a decrease in total cholesterol levels, a decrease in low-density lipoprotein (LDL), very-low-density lipoprotein (VLDL) and triglyceride fractions, as well as an increase in high-density lipoprotein (HDL) fraction. It has also been shown that chronic use of α1-blockers has no effect on the carbohydrate metabolism and increases insulin sensitivity [3].

l-arginine (Arg) is an endogenous α-amino acid produced from citrulline (Citr), primarily in the urea cycle, but also in the so-called citrulline/arginine cycle [4]. Arg is involved in the synthesis of many essential building blocks of the body as well as in regulatory and cell relay. It participates in the formation of proteins, urea, creatine, proline, polyamines (putrescine, spermine, spermidine), and glutamine. Citr, as opposed to Arg, is not involved in protein synthesis, but is essential for toxic ammonium ion removal in the urea cycle [4]. Importantly, Arg also participates in the synthesis of nitric oxide (NO), which in the vascular endothelium serves as a vasodilator factor (EDRF: endothelium-derived relaxing factor). NO activates guanylate cyclase in smooth muscle, which increases the intracellular concentration of cyclic guanosine monophosphate (cGMP) caused by vasodilation. NO action on blood vessels occurs not only directly, but also indirectly through the attenuation of the sympathetic nervous system and the renin inhibition of endothelials (hormones modulating the secretory kidney). Furthermore, NO lowers blood pressure, vascular wall tension, and remodeling, and influences blood vessel wall tension caused by both physiological and pharmacological factors. NO is also a neurotransmitter in the peripheral nervous system and the brain that may be involved in short-term memory processes [4].

Currently, Arg is used in the treatment of many diseases, mainly in urea cycle disorders [5]. However, it is also applied in the treatment and prevention of many cardiovascular system diseases, such as hypercholesterolemia, atherosclerosis, pulmonary hypertension, and chronic renal failure [6]. Clinical trials carried out by Suzuki *et al.* demonstrated that the local administration of Arg after stent implantation, together with an oral anticoagulant (salicylic acid or ticlopidine), prevents restenosis [7]. There is also evidence in the literature that the administration of Arg in hypercholesterolemia reduces platelet aggregation [8]. What is promising is also combinational therapy involving Arg with *N*-acetylcysteine in the treatment of antiatherogenic hypertension, type II diabetes, as well as the prevention of cardiovascular disease in these patients [9].

Biodegradable polymers belong to a class of important and desirable biomaterials because of their wide application in the biomedical field, including tissue engineering, controlled drug delivery, and gene therapy [10,11,12]. Aliphatic polyesters such as polylactide (PLA) and poly(ε-caprolactone) (PCL) are the most commonly applied due to their good biocompatibility, low immunogenicity, and suitable mechanical properties [13,14].

Previously, approaches using prazosin polymeric delivery systems, which allow the therapeutic agent to be targeted to the disease site with minimal systemic effects, have been demonstrated. Li *et al.* synthesized the biodegradable polymer (poly-*N*5-(3-hydroxypropyl-l-glutamine) (PHPG), a prazosin conjugate, by covalent coupling of prazosin to PHPG via a carbamate bond. *In vitro* studies carried out in a phosphate-buffered saline solution (pH 7.4) showed that 0.92 mg of prazosin was released per day at 100 mg of the obtained conjugate [15]. Furthermore, *in vivo* studies performed with New Zealand white rabbits demonstrated a nearly constant plasma prazosin concentration profile above 2 ng/mL, which was maintained for 10 days [15]. The hydrogel membranes of sodium alginate (SA) and poly(vinyl alcohol) (PVA) were prepared by the solvent casting method for transdermal delivery of prazosin hydrochloride [16]. The differential scanning calorimetry (DSC) analysis confirmed the hydrogen membrane formation and suggested that the membrane stiffness increased with increased concentration of glutaraldehyde (GA) in the membranes. The *in vitro* drug release study was performed through rat abdominal skin, showing that the drug release was dependent on the concentrations of GA in membranes [16]. In addition, evaluation of the effect of changing the Eudragit RL 100 ratio and the influence of different penetration enhancers in various concentrations on the release of prazosin from Carboset 525:Eudragit RL 100 polymeric films was performed by Hosny *et al*. [17]. The obtained results show that prazosin release from polymeric films containing Carboset 525:Eudragit RL 100 in a 1:l ratio was significantly higher than from films in a 1:0.25 ratio.

The above studies provide useful information for the development of clinically acceptable transdermal therapeutic systems for prazosin. However, to the best of our knowledge, there is no literature report on the covalent conjugation of prazosin to PCL and PLA for the development of a polymeric implantable drug delivery carrier.

In our previous study, biodegradable polymers were effectively synthesized through a ring-opening polymerization (ROP) of ε-caprolactone (ε-CL) and l,l-lactide (LLA), initiated by natural- and safe for human-health α-amino acids (Arg and Citr). In our current work, these obtained and characterized polymeric matrices was first subjected to cyto- and genotoxicity tests and then covalently bonded to prazosin using the CDI (carbonyl diimidazole) coupling method for the development of a novel implantable macromolecular drug delivery carrier. *In vitro* release rate of the drug from the synthesized biodegradable macromolecular conjugate was analyzed. It was found that the drug release rate was dependent on pH and the average molecular weight of polymeric matrices. Such urethane-linked prazosin conjugates appear to be a promising drug carrier, which could improve the pharmacokinetics of prazosin, as well as increase its efficiency as a result of the synergistic action of prazosin with Arg, which is applied in the treatment and prevention of many cardiovascular system diseases.

## 2. Results and Discussion

In general, drugs conjugated to polymeric carriers can achieve decreased non-specific toxicity, enhanced therapeutic efficacy, a sustained drug release profile, optimized drug biodistribution, increased circulation time, and targeted drug delivery [18].

In the current study, two-arm biodegradable polymeric matrices composed of biodegradable PCL or PLLA with naturally incorporated Arg or Citr were utilized for the covalent conjugation of prazosin as a model α1-blocker for the creation of a new and promising prazosin-macromolecular conjugate.

Owing to the fact that the materials applied in medicine and pharmacy must fulfill the relevant requirements (pharmacopoeia standards), the synthesized polymeric products (PCL/Arg, PCL/Citr, PLLA/Arg, and PLLA/Citr) were first subjected to cyto- and genotoxicity tests. The luminescent bacteria *V. fischeri*, ciliated protozoa *S. ambiguum*, and *Salmonella typhimurium* were used to perform cyto- and genotoxicity assays. It was found that the synthesized polymeric matrices were not cytotoxic to all test bionts; thus, both bacteria and protozoa proved adequate for biomedical applications (Table 1) [19].

**Table 1:** The cytotoxicity results of the synthesized polymeric matrices.

Sample	Microtox 15 min-EC50 ^a^	Microtox 30 min-EC50 ^a^	Spirotox 24 h-EC50 ^a^
**PCL/Arg**	0	0	0
**PLLA/Arg**	0	0	0
**PCL/Citr**	0	0	0
**PLLA/Citr**	0	0	0

^a^ Percent of toxic effect.

Regarding genotoxicity results, the PCL/Arg, PCL/Citr, and PLLA/Citr extracts demonstrated no genotoxic potential for *Salmonella typhimurium* TA1535 in the umu-test performed with or without the addition of s9 fraction. The activity of β-galactosidase for these samples remained at the level of a negative control, indicating no induction of the umuC gene. The weak genotoxic potential to the tested strain was revealed for the PLLA/Arg extract when tested without metabolic activation. The β-galactosidase activity was increased and the induction ratio (IR) was 3.03 ± 0.30 when the extract was diluted 1.5-fold (66.66% concentration of the extract). However, further dilution of the sample (three-fold, six-fold, and 12-fold to the extract concentration of 33.33%, 16.66% ,and 8.33%, respectively) resulted in the complete loss of genotoxic potential (Table 2). Additionally, there was no toxicity (no growth inhibition of the *Salmonella* strain) for the PLLA/Arg extracts.

**Table 2:** The IR value determined for the PLLA/Arg extracts.

**Extract Concentration**	8.33%	16.66%	33.33%	66.66%
**IR**	0.94 ± 0.06	1.11 ± 0.10	1.41 ± 0.03	3.03 ± 0.30

On the other hand, genotoxic activity was completely eliminated if the sample was tested under the condition of metabolic activation with a liver s9 fraction. The activity of β-galactosidase was then compared with the negative control and the IR value never reached the level of 1.5, regardless of the dilution of the PLLA/Arg extract. It can therefore be concluded that the agent occurring in the PLLA/Arg extract and responsible for genotoxic action was inactivated with metabolic activation, which is the natural process that occurs in the human organism.

**Scheme 1:**
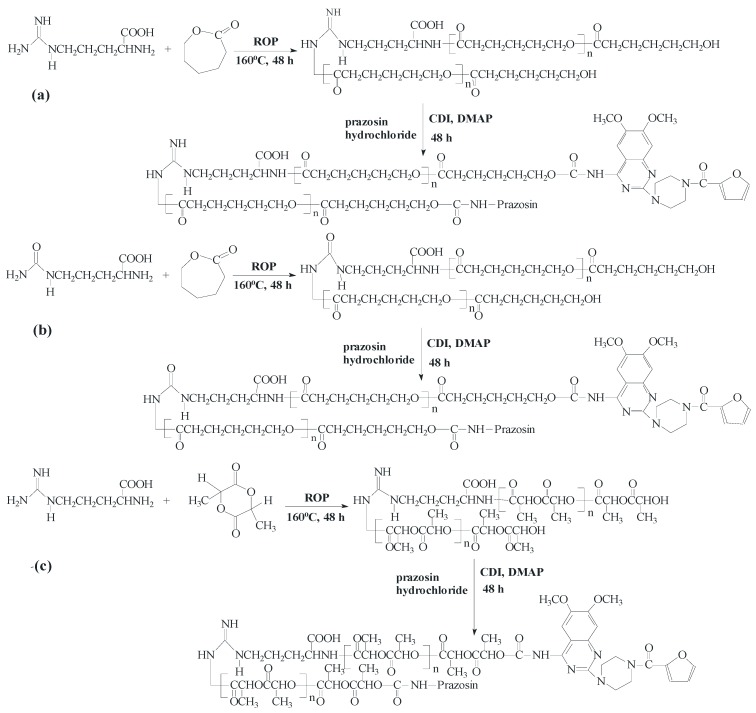

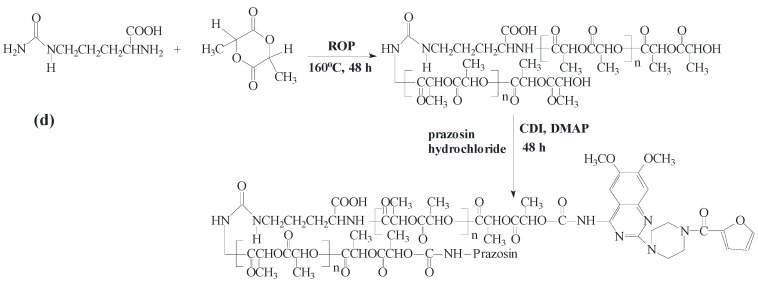
Synthesis of the biodegradable macromolecular conjugates of prazosin ((**a**) prazosin–PCL/Arg); (**b**) prazosin–PCL/Citr; (**c**) prazosin–PLLA/Arg; and (**d**) prazosin–PLLA/Citr).

**Figure 1:**
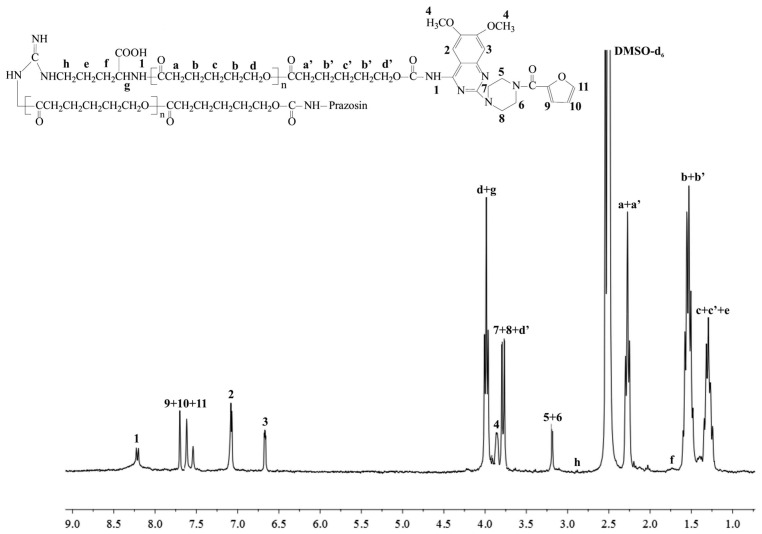
^1^H-NMR spectrum of the prazosin–PCL/Arg conjugate.

**Figure 2:**
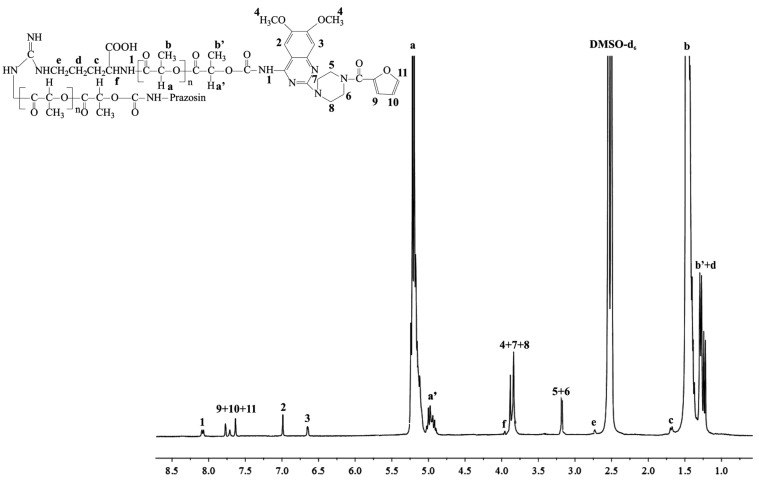
^1^H-NMR spectrum of the prazosin–PLLA/Arg conjugate.

New urethane bonds were formed between the hydroxyl end-groups of the synthesized polymer matrices and amine groups of the drug. CDI was used as a coupling agent in the presence of DMAP as a catalyst (Scheme 1). Figure 1 and Figure 2 show the ^1^H-NMR spectra of the obtained macromolecular conjugates of prazosin (e.g., prazosin–PCL/Arg and prazosin–PLLA/Arg). This technique was also used to estimate drug payloads in the conjugates.

The signal intensities of a newly formed urethane bond (1) (Figure 1 and Figure 2) and the signal intensity of two protons of the methylene group belonging to PCL (a+a′, Figure 1), as well as the protons of the methine group of PLLA (a, Figure 2), were compared, and indicated a 17.4 mol % and 16.9 mol % drug content in the prazosin–PCL/Arg and prazosin–PLLA/Arg conjugates, respectively (calculated as the mole of the drug per mole of the whole macromolecular system) [20] for prazosin–PCL/Citr and prazosin–PLLA/Citr conjugates (see Supplementary Figure S2 and S5). The mole drug content was estimated as 16.3% and 15.8%.

The spectra of the obtained conjugates (Figure 1 and Figure 2) revealed characteristic signals of prazosin, indicating the effective synthesis of covalent biodegradable polyester–prazosin conjugates (yields of conjugation were around 70%). Furthermore, the appearance of a new signal 1, denoted as a signal of a newly formed urethane bond between the hydroxyl end groups of polymer matrices and the amine groups of the drug, confirmed the covalent nature of the obtained polymeric products and discriminated against a simple electrostatic interaction of prazosin with polymer matrices (for comparison, see the ^1^H-NMR spectra of pure prazosin; see Supplementary Figure S7).

### Prazosin Release from the Synthesized Macromolecular Conjugates

The *in vitro* release study was conducted in different buffer solutions with a pH of 1.00 ± 0.05 and 7.00 ± 0.05 at about 37 °C for 28 days (the same study was also conducted at pH 7.4 ± 0.5 (1 M) as in comparison to pH 7.00 ± 0.05, see Supplementary Figure S8). This study allowed us to determine the suitability of the synthesized conjugates and evaluate the potential location in the human body of the implants composed of obtained conjugates.

**Figure 3:**
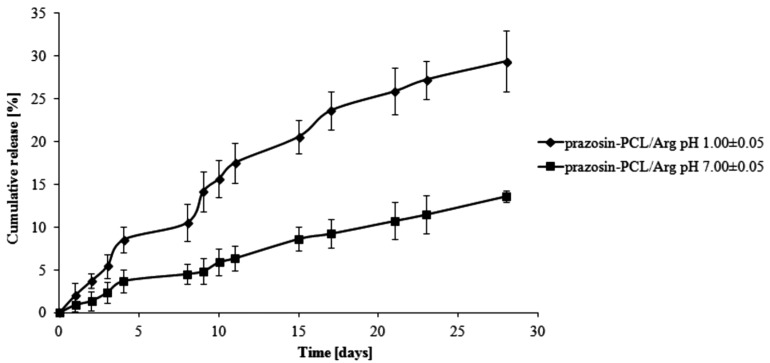
Release profile of prazosin from the prazosin–PCL/Arg conjugate (pH 7.00 ± 0.05 and pH 1.00 ± 0.05).

**Figure 4:**
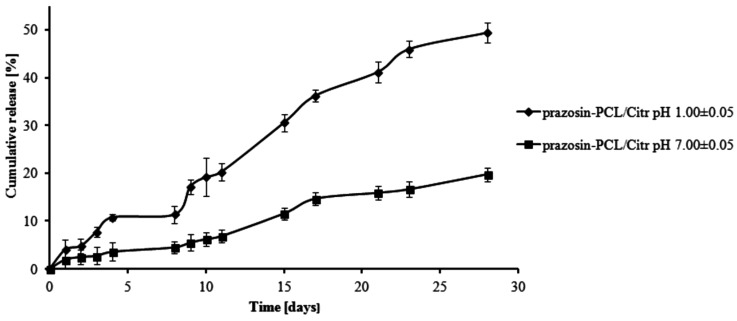
Release profile of prazosin from the prazosin–PCL/Citr conjugate (pH 7.00 ± 0.05 and pH 1.00 ± 0.05).

The total percentage of the prazosin released for prazosin–PCL/Arg (pH 1.00 ± 0.05) was about 2% by weight on the first day of the study. On the third day, this changed to about 5% (see Figure 3). This occurrence was likely due to the release of prazosin from the amorphous phase of the PCL/Arg matrix. After eight days of the study had passed (about 11% of prazosin had now been released), there was a rapid increase in the amount of the drug released, *i.e.*, 21% over 15 days of the study and then 30% on the last day of the study. In the case of the release profile of prazosin from the prazosin–PCL/Arg at pH 7.00 ± 0.05, about 5% of the drug was released following eight days of the study. In the days following, the percentage of the drug released gradually increased and reached a value of 9% and 14% by weight after 15 and 28 days, respectively. The profile of prazosin release from the PCL/Citr matrices was therefore characterized by a high release rate (see Figure 4) and demonstrated that about 31% and 50% of the drug was released after 15 and 28 days of incubation (pH 1.00 ± 0.05), whereas at pH 7.00 ± 0.05, values of 11% and 20% by weight were reached (see Figure 4).

**Figure 5:**
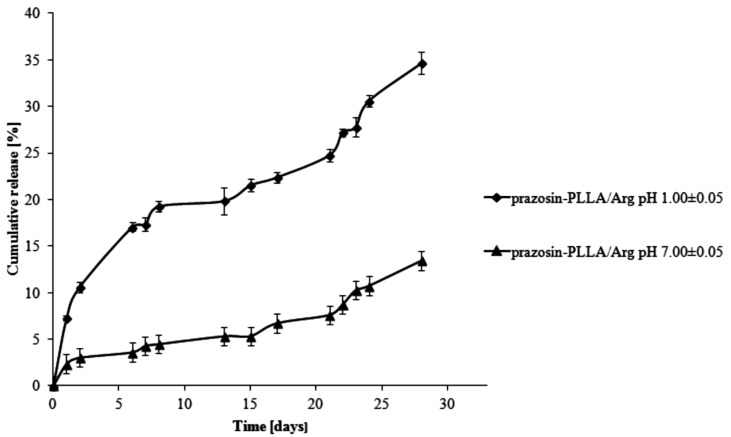
Release profile of prazosin from the prazosin–PLLA/Arg conjugate (pH 7.00 ± 0.05 and pH 1.00 ± 0.05).

**Figure 6:**
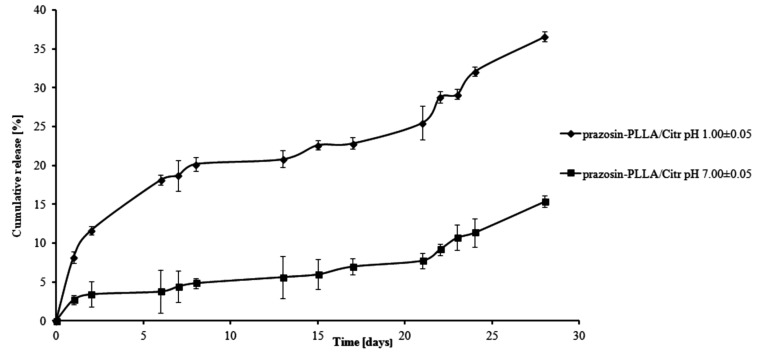
Release profile of prazosin from the prazosin–PLLA/Citr conjugate (pH 7.00 ± 0.05 and pH 1.00 ± 0.05).

For PLLA-based matrices, the percentage of prazosin released was approximately 7% by weight after one day of study (pH 1.00 ± 0.05) and rose vigorously at six days, when it achieved 17% by weight (see Figure 5). In the following days (day eight to day 13 of the study), a visible plateau in the release profile was achieved and at this stage, the amount of the drug released was maintained at about 20%. In the following days, the amount of prazosin released increased, but not as rapidly as it had at the beginning, reaching a value of approximately 35% on day 28 of incubation. The release profile of prazosin at pH 7.00 ± 0.05 differed from its profile at an acidic pH. On the first day of the incubation, the percentage of prazosin released was about 2%. In the following days, a plateau in the release profile was observed (see Figure 5) that was maintained until day 21 of the study and did not exceed 8% prazosin released by weight. Following on, the amount of drug released gradually increased and achieved approximately 13% after 28 days of study. For PLLA/Citr-based matrices, the prazosin release profiles were comparable; after 13 days of incubation, the amount of the drug released was about 21% and on the last day, this value was approximately 37% by weight (pH 1.00 ± 0.05; see Figure 6). In the case of a neutral pH, 7% of the drug was released after 13 days of study, while after 28 days, this value reached approximately 15%.

The observed differences between the release profiles of prazosin from PCL- and PLLA-based matrices result from the different physicochemical natures of the polymers and their different average molecular weight, when used to synthesize prazosin conjugates. Although PCL-based matrices are inherently more hydrophobic, the release profiles of prazosin from the PCL/Citr matrix were more intense than for PLLA. This was due to differences in the average molecular weight of applied polymers. The average molecular weight of the PCL/Arg matrix was about 5780 g/mol, whereas for PCL/Citr it was 3840 g/mol. Therefore, about 30% of prazosin was released after 28 days of incubation for the prazosin–PCL/Arg conjugate and about 50% for the prazosin–PCL/Citr conjugate at pH 1.00 ± 0.05 (Figure 3 and Figure 4). In the case of PLLA-based conjugates, the average molecular weight was similar (6250 g/mol for the synthesis of the prazosin–PLLA/Arg conjugate and 6800 g/mol for the prazosin–PLLA/Citr conjugate), therefore the percentage of prazosin released for these two cases was approximately the same (35% for prazosin–PLLA/Arg and 37% for prazosin–PLLA/Citr at acidic pH; Figure 5 and Figure 6). Our current results are in an agreement with those obtained by our group previously [21] and clearly confirm that the average molecular weight of the matrices has a significant effect on the total percentage of the drug released.

The stability of prazosin before, during, and after the *in vitro* release tests was maintained using the HPLC-UV method. For instance, see the HPLC chromatogram and UV spectrum of prazosin released from the prazosin–PCL/Arg conjugate (see Supplementary Figure S9). As we can easily see, the drug release was stable during and after testing, and thus adequate for further applications.

As is known, the hydrolytic stability of polymers depends on many factors, such as the crystallinity, size and form of the crystallites, their morphological structure, *etc*.

**Figure 7:**
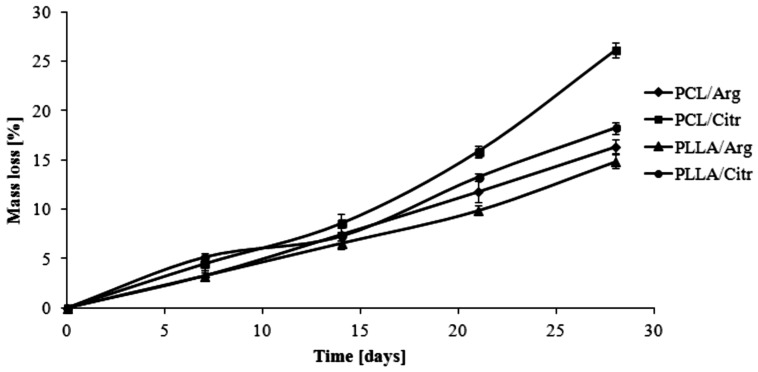
Effect of hydrolytic biodegradation time on the *WL* of polymeric matrices.

The hydrolytic degradation of the obtained polymeric matrices was investigated by monitoring their *WL* and the results are shown in Figure 7. The *WL* values of the PCL/Arg matrix achieved approximately 16% after 28 days of degradation, whereas 26% for the PCL/Citr matrix was achieved at the same time of study. The matrices based on PLLA were characterized by hydrolytic stability similar to PCL/Arg and the *WL* in these two cases was about 14% and 18% for PLLA/Arg and PLLA/Citr, respectively. The results were directly compared to the results of the intrinsic viscosity measurements (*η_inh_*), which follow the same trend (Table 3) [22]. Furthermore, they were well correlated with the percentage of prazosin released, thereby confirming the dependence of the average molecular weight of the matrices on the release profile of the covalent attached drug.

The thermal behaviour of the polymeric conjugates is important in terms of their properties pertaining to controlled drug release and their processing into suitable dosage forms [23].

**Figure 8:**
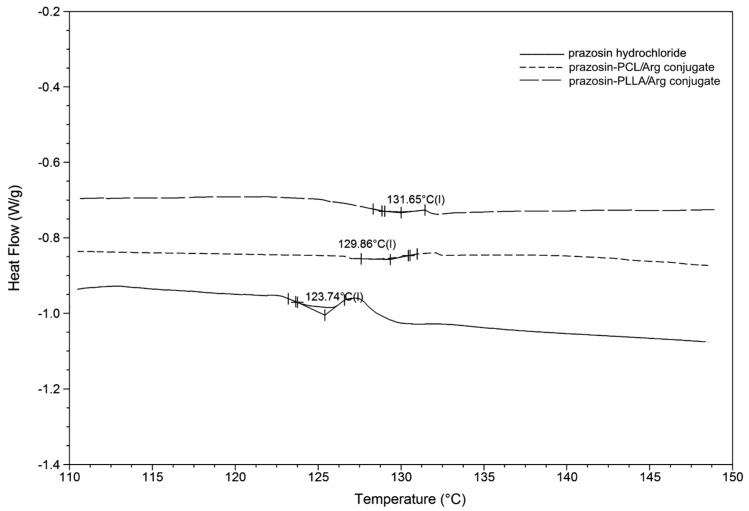
MDSC thermogram of a pure prazosin hydrochloride and synthesized macromolecular conjugates of prazosin.

Modulated differential scanning calorimetry (MDSC) was used to determine the glass transition temperature (T_g_) value of the obtained prazosin–polymeric conjugates compared to pure prazosin hydrochloride (see Figure 8). Higher T_g_ is generally a favorable property, since compounds with high T_g_ have a reduced ability to recrystallize at a given temperature, compared to those that have a lower T_g_. MDSC analysis indicated that the synthesized macromolecular conjugates showed higher T_g_ compared to the resulting T_g_ value of pure prazosin (T_g_ values of polymer chains were 63.1 °C for PLLA and −64.8 °C for PCL) [24]. These results were attributed to the rigid prazosin pendant groups, which decrease the mobility of polymer chains, a fact supported in the literature [25].

**Table 3:** The hydrolytic degradation results of the synthesized polymeric matrices.

Time (days)	PCL/Arg			PCL/Citr			PLLA/Arg			PLLA/Citr		
	*WL* (%)	*η_inh_* ^a^	Δ *η_inh_* (%)	*WL* (%)	*η_inh_* ^a^	Δ *η_inh_* (%)	*WL* (%)	*η_inh_* ^a^	Δ *η_inh_* (%)	*WL* (%)	*η_inh_* ^a^	Δ *η_inh_* (%)
**7**	3.3±0.8	0.10	9	4.5 ± 0.4	0.07	13	3.2 ± 0.2	0.27	3	5.1 ± 0.2	0.28	6
**14**	7.5 ± 0.4	0.10	9	8.6 ± 0.5	0.07	13	6.6 ± 0.6	0.26	6	7.3 ± 0.3	0.27	9
**21**	11.8 ± 0.8	0.09	18	15.8 ± 0.9	0.06	25	9.8 ± 0.9	0.24	12	13.3 ± 0.9	0.25	14
**28**	16.4 ± 0.6	0.09	18	26.1 ± 0.2	0.06	25	14.9 ± 0.8	0.23	15	18.2 ± 0.6	0.23	20

^a^ The *η_inh_* was determined by a viscosity method. The *η_inh_* of the synthesized polymeric matrices before degradation was: 0.11 (dL/g) for PCL/Arg; 0.08 (dL/g) for PCL/Citr; 0.28 (dL/g) for PLLA/Arg and 0.30 (dL/g) for PLLA/Citr; The Δ*η_inh_* was decrease of *η_inh_*.

## 3. Experimental Section

### 3.1. Materials

Prazosin hydrochloride (1-(4-amino-6,7-dimethoxy-2-quinazolinyl)-4-(2-furanylcarbonyl)piperazine hydrochloride, >98.0%, TCI EUROPE N.V., Zwijndrecht, Belgium), 1,1′-Carbonyldiimidazole (CDI, ≥97.0%, Sigma Co., Poznan, Poland) and 4-(dimethylamino)pyridine (DMAP, ≥99%, Aldrich Co., Poznan, Poland) were used without further purification. Dichloromethane (DCM, anhydrous, ≥99.8%, POCh, Gliwice, Poland), diethyl ether (anhydrous, ≥99.7%, POCh, Gliwice, Poland) and dimethyl sulphoxide (DMSO, anhydrous, 99%, Aldrich Co.) were used as received. Buffer solutions: pH 7.00 ± 0.05, 0.1 M (potassium dihydrogen phosphate/di-sodium hydrogen phosphate, 20 °C), pH 1.00 ± 0.05, 0.1 M (glycine buffer, 20 °C), and pH 7.4 ± 0.5, 1 M, Avantor Performance Materials S.A., Gliwice, Poland) were also used as received.

### 3.2. Methods

#### 3.2.1. Synthesis of Macromolecular Conjugates of Prazosin

The biodegradable matrices (denoted as PCL/Arg, PCL/Citr, PLLA/Arg, and PLLA/Citr) based on polyesters (PCL and PLLA) and α-amino acids (Arg and Citr) were applied to the preparation of prazosin-macromolecular conjugates. All of the reactions were carried out in an argon atmosphere. The synthesis and physicochemical properties of the synthesized matrices were described in our previous paper [26].

#### 3.2.2. Synthesis of Prazosin-Conjugated PCL/Arg and PCL/Citr

The synthesized matrices (PCL/Arg or PCL/Citr, their average molecular weight determined by GPC: 5780 g/mol (400 mg, 69 µmol) or 3840 g/mol (400 mg, 104 µmol) [26], respectively, were dissolved in 4 mL of anhydrous DMSO. CDI (39 mg, 242 µmol for PCL/Arg or 59 mg, 364 µmol for PCL/Citr) was dissolved in 2 mL of anhydrous DMSO and added dropwise to the appropriate polymer solution. The reaction mixture was stirred for 24 h at room temperature for the activation of polymers. Prazosin hydrochloride (73 mg, 173 µmol for PCL/Arg or 109 mg, 260 µmol for PCL/Citr) and DMAP (44 mg, 363 µmol for PCL/Arg or 67 mg, 546 µmol for PCL/Citr) were firstly dissolved in 4 mL of anhydrous DMSO. Then, the received solutions were dropped into appropriate CDI-activated polymer solutions with stirring at 40 °C for 24 h in a nitrogen atmosphere. After the coupling reaction, the reaction mixtures were precipitated twice in an excess of cold diethyl ether and dried for 24 h *in vacuo*. The received products were then purified using dialysis membrane (MWCO = 500) for 24 h to remove the unreacted prazosin hydrochloride, DMAP, and CDI. Dialyzed products were dried for 48 h *in vacuo* to eliminate the residual solvents and then analyzed by 1H-NMR and FTIR (the conjugate yields: 74% for prazosin–PCL/Arg and 70% for prazosin–PCL/Citr).

*Prazosin–PCL/Arg*: ^1^H-NMR (DMSO-*d*_6_, 300 MHz, δ_H_, ppm): 1.24–1.32 (m, -CH_2_C**H**_2_CH_2_- of PCL (c+c′) and -C**H**_2_CH_2_CH-(COOH)- of Arg (e), 1.48–1.57 (m, -C**H**_2_CH_2_C(O)- of PCL (b+b′), 1.71 (m, -CH_2_C**H**_2_CH-(COOH)- of Arg (f), 2.25–2.29 (t, -CH_2_C**H**_2_C(O)- of PCL (a+a′)), 2.87 (m, NH-C**H**_2_CH_2_- of Arg (h), 3.18 (s, piperazine (5 and 6)), 3.73–3.78 (m, piperazine (7 and 8) and -C**H**_2_O-C(O)-NH- of PCL (d′)), 3.88 (s, methoxy groups of chinazolin (4)), 3.96–4.01 (m, -C**H**(-COOH)NH- of Arg (g) and -C**H**_2_O- of PCL (d), 6.67 (s, chinazolin (3)), 7.06 (s, chinazolin (2)), 7.49–7.88 (m, furan (9, 10, and 11)), 8.21 (m, -CH_2_O-C(O)-N**H**- (1) (see Figure 1).

FTIR (KBr, cm^−1^): 3437–3080 (υ_N-H_ and υ_C-H aromatic_), 2946–2866 (υ*_as_*_CH2_), 1726 (υ_C=O_), 1670–1641 (υ_C=O_) 1585–1560 (δ_N-H_), 1472 (υ_C-H_), 1368 (δ*_as_*_CH3_), 1294 (υ_C-O_ and _C-C_), 1242 (υ*_as_*_COC_), 1186 (υ_OC-O_), 1046 (υ_C-O-C_) (see Supplementary Figure S1).

*Prazosin–PCL/Citr*: ^1^H-NMR (DMSO-*d*_6_, 300 MHz, δ_H_, ppm): 1.24–1.35 (m, -CH_2_C**H**_2_CH_2_- of PCL (c+c′) and -C**H**_2_CH_2_CH-(COOH)- of Citr (e)), 1.45–1.58 (m, -CH_2_C**H**_2_C(O)- of PCL (b+b′)), 1.74 (m, -CH_2_C**H**_2_CH-(COOH)- of Citr (f)), 2.25–2.30 (t, -CH_2_C**H**_2_C(O)- of PCL (a+a′)), 2.89 (m, NH-CH_2_C**H**_2_- of Citr (h)), 3.18 (s, piperazine (5 and 6)), 3.74–3.90 (m, piperazine (7 and 8) and -C**H**_2_O-C(O)-NH- of PCL(d′)), 3.84 (s, methoxy groups of chinazolin (4)), 3.95–4.01 (m, -C**H**(-COOH)NH- of Citr (g) and -C**H**_2_O- of PCL (d), 6.67 (s, chinazolin (3)), 7.08 (s, chinazolin (2)), 7.50–7.89 (m, furan (9, 10, and 11)), 8.22 (m, -CH_2_O-C(O)-N**H**- (1) (see Supplementary Figure S2).

FTIR (KBr, cm^−1^): 3437–3022 (υ_N-H_ and υ_C-H aromatic_), 2946–2866 (υ*_as_*_CH2_), 1750–1728 (υ_C=O_), 1640 (υ_C=O_) 1585–1560 (δ_N-H_), 1472 (υ_C-H_), 1368 (δ*_as_*_CH3_), 1294 (υ_C-O_ and _C-C_), 1242 (υ*_as_*_COC_), 1142 (υ_OC-O_), 1046 (υ_C-O-C_) (see Supplementary Figure S3).

#### 3.2.3. Synthesis of Prazosin-Conjugated PLLA/Arg and PLLA/Citr

The synthesized matrices (PLLA/Arg or PLLA/Citr, average molecular weight determined by GPC: 6250 g/mol (400 mg, 64 µmol) or 6800 g/mol (400 mg, 59 µmol) [26], respectively, were dissolved in 4 mL of anhydrous DMSO. CDI (36 mg, 224 µmol for PLLA/Arg or 33 mg, 206 µmol for PLLA/Citr) was dissolved in 2 mL of anhydrous DMSO and added dropwise to the appropriate polymer solution. The reaction mixture was stirred for 24 h at room temperature for the activation of polymers. Prazosin hydrochloride (67 mg, 160 µmol for PLLA/Arg or 62 mg, 147 µmol for PLLA/Citr) and DMAP (41 mg, 336 µmol for PLLA/Arg or 38 mg, 309 µmol for PLLA/Citr) were firstly dissolved in 4 mL of anhydrous DMSO. Then, the received solutions were dropped into appropriate CDI-activated polymer solutions with stirring at 40 °C for 24 h in a nitrogen atmosphere. After the coupling reaction, the reaction mixtures were precipitated twice in an excess of cold diethyl ether and dried for 24 h *in vacuo*. The received products were then purified using dialysis membrane (MWCO = 500) for 24 h to remove the unreacted prazosin hydrochloride, DMAP and CDI. Dialyzed products were dried for 48 h *in vacuo* to eliminate the residual solvents and then analyzed by 1H NMR and FTIR (the yields: 66% for prazosin–PLLA/Arg and 71% for prazosin–PLLA/Citr).

*Prazosin–PLLA/Arg*: ^1^H-NMR (DMSO-*d*_6_, 300 MHz, δ_H_, ppm): 1.21–1.29 (m, -C**H**_3_ of PLLA (b′) and -C**H**_2_CH_2_CH-(COOH)- of Arg (d)), 1.46 (d, -C**H**_3_ of PLLA (b)), 1.71 (m, -CH_2_C**H**_2_CH-(COOH)- of Arg (c)), 2.74 (m, NH-C**H**_2_CH_2_- of Arg (e)), 3.11 (s, piperazine (5 and 6)), 3.79–3.84 (m, piperazine (7 and 8) and methoxy groups of chinazolin (s, 4), 4.01 (m, -CH(-COOH)NH- of Arg (f)), 4.89–5.00 (m, -C**H**(CH_3_)- of PLLA (a′)), 5.12–5.24 (q, -C**H**(CH_3_)- of PLLA (a)), 6.65 (s, chinazolin (3)), 7.03 (s, chinazolin (2)), 7.59–7.86 (m, furan (9, 10, and 11)), 8.17 (m, -CH_2_O-C(O)-N**H**- (1)) (see Figure 2).

FTIR (KBr, cm^−1^): 3343(υ_N-H_), 2996–2945 (υ*_as_*_CH2_), 1758 (υ_C=O_), 1649 (υ_C=O_) 1550–1532 (δ_N-H_), 1456 (υ_C-H_), 1368-1360 (δ*_as_*_CH3_), 1261 (υ*_as_*_COC_), 1134 (υ_OC-O_), 1046 (υ_C-O-C_) 955 _(rCH3+νC-C),_ 755 (δ_N-H_) (see Supplementary Figure S4).

*Prazosin–PLLA/Citr*: ^1^H-NMR (DMSO-*d*_6_, 300 MHz, δ_H_, ppm): 1.22–1.29 (m, -C**H**_3_ of PLLA (b′) and -C**H**_2_CH_2_CH-(COOH)- of Citr (d)), 1.39–1.46 (d, -C**H**_3_ of PLLA (b)), 1.70 (m, -CH_2_C**H**_2_CH-(COOH)- of Citr (c)), 2.72 (m, NH-C**H**_2_CH_2_- of Citr (e)), 3.18 (s, piperazine (5 and 6)), 3.78–3.84 (m, piperazine (7 and 8) and methoxy groups of chinazolin (s, 4)), 4.00 (m, -C**H**(-COOH)NH- of Citr (f)), 4.88–4.98 (m, -C**H**(CH_3_)- of PLLA (a′)), 5.12–5.24 (q, -CH(CH_3_)- of PLLA (a)), 6.66 (s, chinazolin (3)), 7.08 (s, chinazolin (2)), 7.54–7.88 (m, furan (9, 10, and 11)), 8.20 (m, -CH_2_O-C(O)-N**H**- (1)) (see Supplementary Figure S5).

FTIR (KBr, cm^−1^): 3443–3100 (υ_N-H_ and υ_C-H aromatic_), 2996–2945 (υ*_as_*_CH2_), 1755 (υ_C=O_), 1649 (υ_C=O_), 1545–1532 (δ_N-H_), 1456 (υ_C-H_), 1384–1360 (δ*_as_*_CH3_), 1261 (υ*_as_*_COC_), 1134 (υ_OC-O_), 1046 (υ_C-O-C_), 955 _(rCH3+νC-C),_ 755 (δ_N-H_) (see Supplementary Figure S6).

#### 3.2.4. Toxicity Assays

A Microtox^®^ assay with the luminescent bacteria *Vibrio fischeri* was performed with the lyophilized bacteria purchased from Modern Water (New Castle, PA, USA). The test was performed using disposable glass cuvettes. As a diluent and a control, 2% NaCl containing a 20 mM Tris buffer (pH 7.4) was used. Samples were incubated at 15 °C for 15 and 30 min and the light output of the samples was recorded with a Microtox^®^ M500 analyser.

A Spirotox test with the ciliate protozoan *Spirostomum ambiguum* was performed according to the standard protocol [27]. The test was carried out in 24 disposable, polystyrene microplate wells. As a diluent and a control, a Tyrode’s solution was used. Ten organisms were added to each of the microplate wells. The samples were incubated in the dark at 25 °C for 24 h. Following on, the test responses, *i.e.*, the different deformations of the cell and lethal responses, were observed with the use of a dissection microscope. Then, 10 mg of the polymer was extracted with 10 mL of Tyrode’s solution at 37 °C for 24 h. Prior to the toxicity test, the extract was neutralized with 0.1 M NaOH. Then, 1 mL of 0.4 M Tris (pH 7.4) was added and the Tyrode’s solution was poured up to 20 mL. One milliliter of the extract refers to 0.5 mg of the polymer. All samples were run in triplicate.

#### 3.2.5. The *Umu*-Test

The *umu*-test is a short-term bacterial test for genotoxicity assessment. The test employs the *Salmonella typhimurium* TA1535 strain. The test was carried out in 96-well microplates with and without the metabolic activation by s9 fraction and according to ISO guidelines [28].

An S9 fraction was prepared from the livers of male Sprague-Dawley rats, pre-treated with Aroclor 1254 (500 mg kg^−1^) five days prior to isolation. The test strain was able to respond to different types of DNA damage and was therefore able to detect different types of genotoxins. The genotoxic potential of the sample was measured as the induction of the umuC gene, which is included in the SOS system in bacterial cells. The test strain is genetically changed; as the umuC gene fuses with the lacZ gene (structural gene for β-galactosidase) and the normal lacZ region is deleted, the induction of the umuC gene is assessed as the determination of β-galactosidase activity in a simple colorimetric assay. Induction ratio (IR) is calculated to show the genotoxic potency of the tested sample in the form of the ratio of β-galactosidase activity evaluated for the sample to the negative control. IR ≥ 1.5 was considered as the threshold at which the sample demonstrated genotoxic activity. The assay was quantitative and the dose-response curves presented in a linear region. Additionally, the growth factor (G) of bacteria was calculated to verify the acceptable level of cytotoxicity of the samples. The tested polymers were incubated for 24 h at 37 °C in a buffer solution (1 mg·mL^−1^) and such an extract was investigated in the test. The bacterial strain was exposed to the test samples of 8.33%, 16.66%, 33.33%, and 66.66% concentration extracts. The exposition was carried out for 2 h at 37 °C, with and without the metabolic activation using s9 rats’ liver fractioning. Deionized sterile water was used as a negative control and 4-nitroquinoline-*N*-oxide and 2-aminoanthracene as positive controls.

#### 3.2.6. *In Vitro* Prazosin Release Studies

The *in vitro* release study of prazosin was performed according to the pharmacopeia standard method [29]. It was performed to measure the concentration of prazosin released at pH 7.00 ± 0.05 (using a 0.1 M phosphate buffer solution) and at pH 1.00 ± 0.05 (using a 0.1 M glycine buffer solution). All experiments were carried out in triplicate; 100 mg of dried macromolecular conjugates (prazosin–PCL/Arg, prazosin–PCL/Citr, prazosin–PLLA/Arg, or prazosin–PLLA/Citr) were immersed in 10 mL buffer solutions (pH 7.00 ± 0.05 or 1.00 ± 0.05) and incubated at 37 °C with continuous orbital rotation at 50 cycles/min. At predetermined time intervals, 10-mL samples were withdrawn from the release medium using the filter and then replaced with 10 mL of fresh buffer solution. The absorption of the buffer solution was determined by a UV-Vis spectrophotometer at the absorbance peak with a wavelength at 247 nm [29]. The absorbance peak correlated well with the prazosin concentration. A linear calibration curve was obtained by measuring the absorption of solutions with predetermined prazosin concentrations. The absorbance readings were within the calibration range for all the measurements in this work.

#### 3.2.7. Hydrolytic Degradation

The hydrolytic degradation of the synthesized polymeric matrices (PCL/Arg, PCL/Citr, PLLA/Arg, and PLLA/Citr) was performed in a 10 mL phosphate buffer solution (pH 7.00 ± 0.05) at 37 °C for 28 days. Following hydrolysis, the polymer samples were washed intensively with distilled water to remove any residual buffer solution, followed by drying under reduced pressure for four days. The degradation rates were estimated by weight loss (*WL*, (*%*)), calculated with the following equation:

*WL* (*%*) = 100 × (*W*_0_ − *W_t_*)/*W*_0_

where, *W*_0_ = initial weight and *W_t_* = weight after degradation.

Furthermore, there results were evaluated by the *η_inh_* of the matrices decreasing. The polymeric matrices viscosity was measured in *N*,*N*-dimethylformamide (DMF, at 30 °C) using an Ubbelohde viscometer (on Stabinger Viscometer SVM 3000).

### 3.3. Characterization Techniques

The polymerization products were characterized in the DMSO-*d*_6_ solution by means of ^1^H-NMR (Varian 300 MHz, LabX, Midland, ON, Canada). The FTIR spectra were measured using KBr pellets (Perkin–Elmer spectrometer, PerkinElmer, Rodgau, Germany). The amount of released prazosin was quantitatively determined by UV-Vis spectrophotometry (UV 1202 Shimadzu, Shimadzu Europe GmbH, Duisburg, Germany) in aqueous buffered solutions at the absorption maximum of the free drug (λ = 247 nm) and using a 1-cm quartz cell.

A modulated differential scanning calorimetry technique (MDSC, TA Instruments, New Castle, PA, USA) was used to analyze the thermal transitions of the polymeric conjugates of prazosin and a free drug. The samples were held at 20 °C for 3 min to reach their equilibrium state and were then heated to 200 °C at a rate of 20 °C/min to erase their thermal histories. To record the crystallization curve, the sample was cooled down to 25 °C at a constant rate of 5 °C/min and kept at this temperature for 60 s to allow for the completion of the crystallization. The sample was then heated to 200 °C at a constant rate of 5 °C/min in order to record the melting curve. All the procedures were performed in a dry nitrogen gas atmosphere.

HPLC analysis of prazosin was performed on a Phenomenex-RPC18 column (250 × 4.6 mm, 5 µm) (Dionex P-580 chromatograph, LabX). The eluent was a mixture of water, acetonitrile, and acetic acid (61%:38%:1%). The prazosin was spectrophotometrically detected at 247 nm.

## 4. Conclusions

In this study, the polymeric conjugates of prazosin were obtained by covalent conjugation of the drug to biodegradable matrices based on PCL or PLLA and natural α-amino acids (Arg and Citr) for the obtaining of polymeric implantable drug delivery carrier. The resulted polymer matrices were first subjected to cyto- and genotoxicity testing (bacterial luminescence test, protozoan assay, and *Salmonella typhimurium* TA1535), which confirmed their non-toxicity and thus the possibility of their application in medicine and pharmaceuticals. The presence of a newly formed urethane bond between the drug and the macromolecular matrices was confirmed by spectral analysis. Furthermore, DSC thermodiagrams of the conjugates indicated that the presence of prazosin pendant groups in the macromolecule structures increased the polymer’s rigidity alongside increasing glass transition temperature. The *in vitro* release profile of prazosin was then examined at two different pH levels, similar to the conditions present in the human body. This study determined the effectiveness of the synthesized conjugates and rejected conclusions about their further application potential. It was found that the release profile of prazosin was dependent on the properties of the matrices used, particularly their average molecular weight. At the same time, the hydrolytic biodegradation of polymer matrices was carried out and yielded up to 26% weight loss over 28 days. It is also important to note that the synthesized conjugates were based on pharmacologically-active and natural α-amino acids (Arg and Citr), compounds applied in the treatment and prevention of many cardiovascular system diseases. Therapeutic efficiency and patient compliance can therefore be improved using the synergistic effect of prazosin and incorporated amino acids.

## Supplementary Materials

Supplementary materials can be accessed at:  http://www.mdpi.com/1420-3049/20/08/14533/s1.
