# Supplementary material for: Prazosin-Conjugated Matrices Based on Biodegradable Polymers and α-Amino Acids—Synthesis, Characterization, and in Vitro Release Study
Source: Molecules. 2015 Aug 12;20(8):14533–51. doi: 10.3390/molecules200814533 (PMC6332215; doi:10.3390/molecules200814533)
Supplement: Supplementary file 1 [file molecules-20-14533-s001.pdf]

Supplementary Information

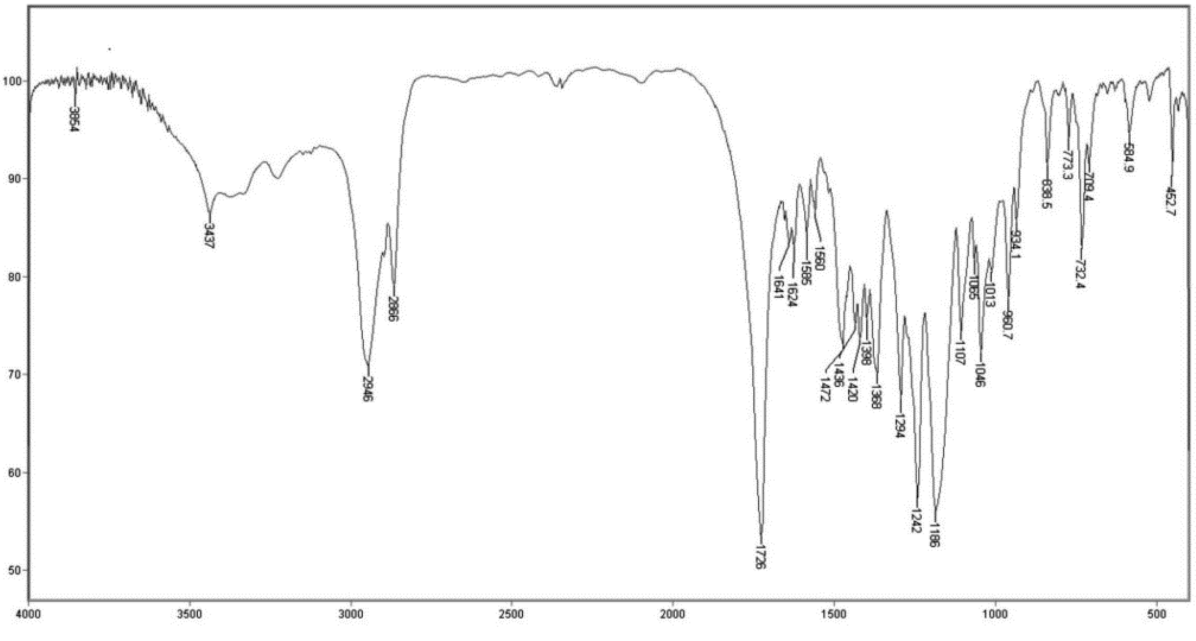

Figure S1. FTIR spectrum of prazosin-PCL/Arg conjugate.

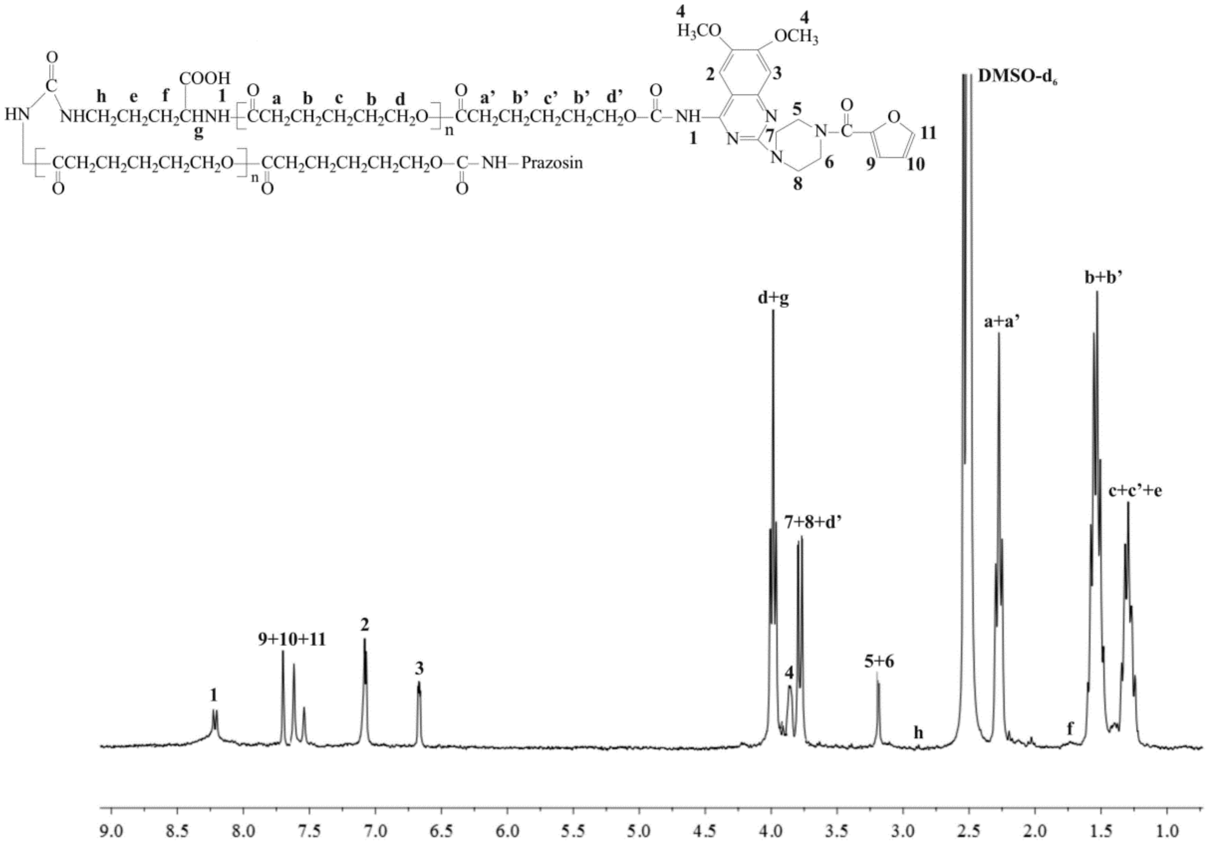

Figure S2. ^{1}H NMR spectrum of the prazosin-PCL/Citr conjugate.

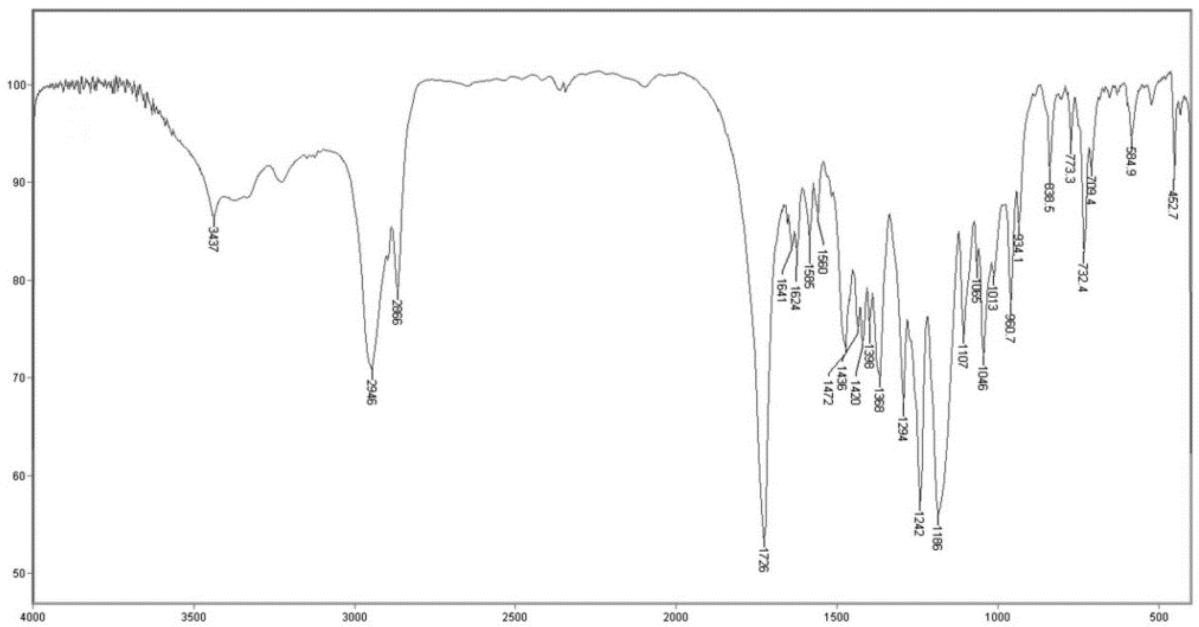

Figure S3. FTIR spectrum of prazosin-PCL/Citr conjugate.

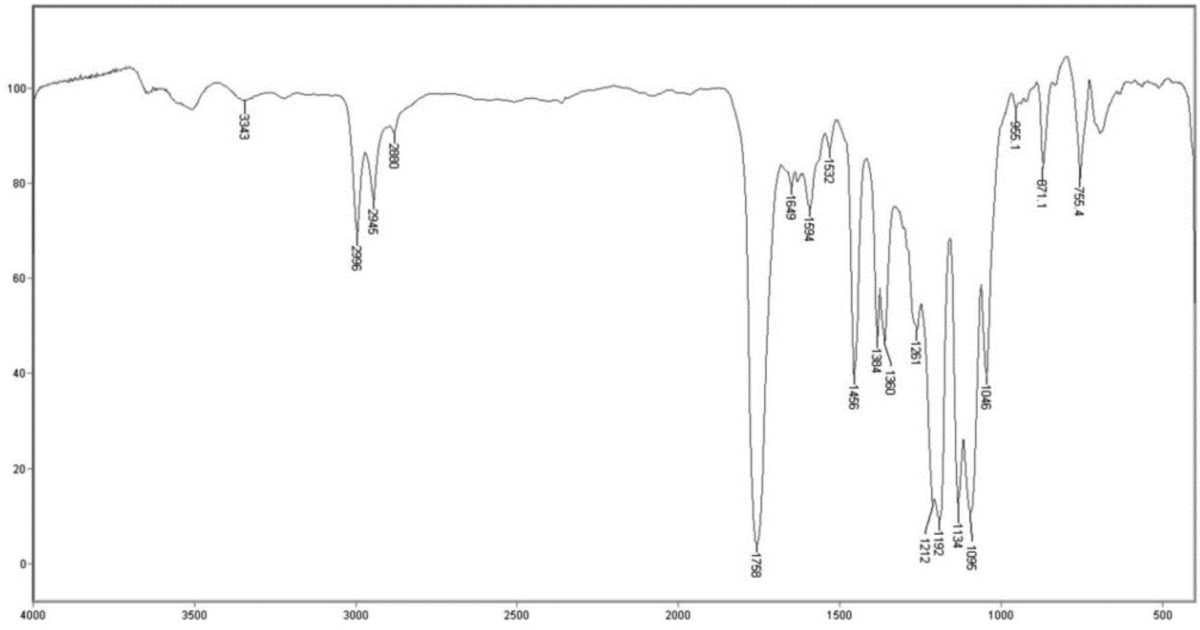

Figure S4. FTIR spectrum of prazosin-PLLA/Arg conjugate.

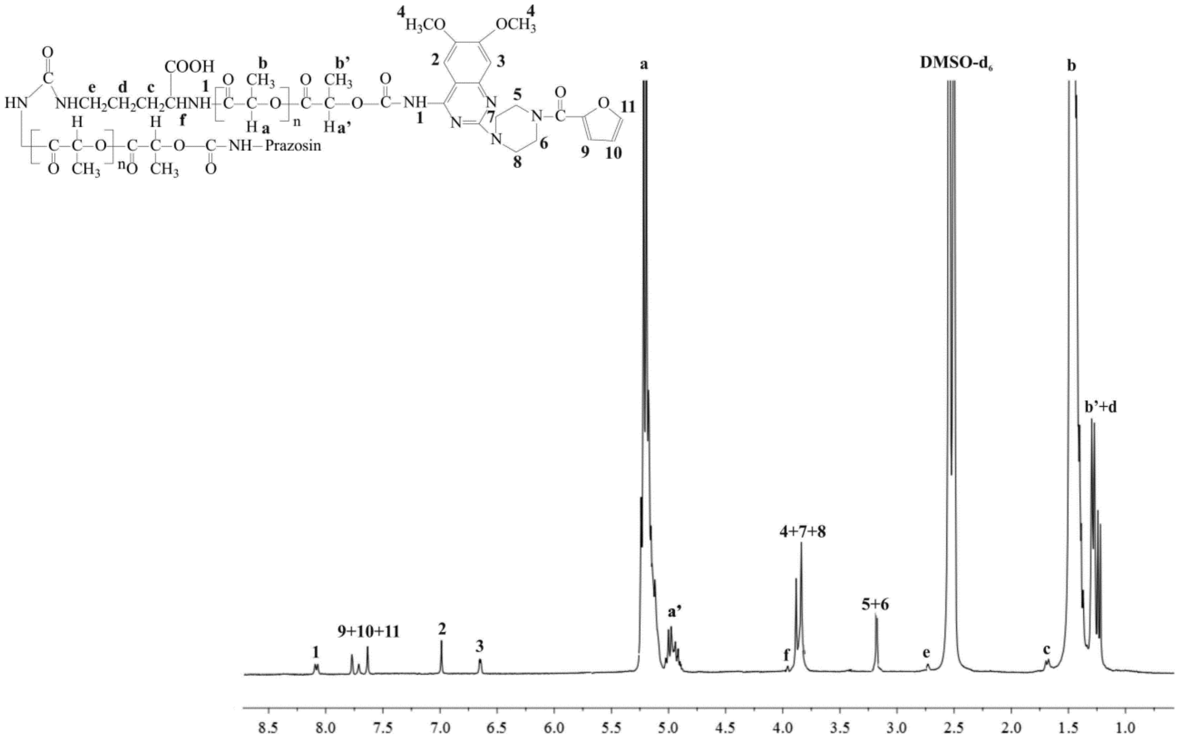

Figure S5. ^{1}H NMR spectrum of the prazosin-PLLA/Citr conjugate.

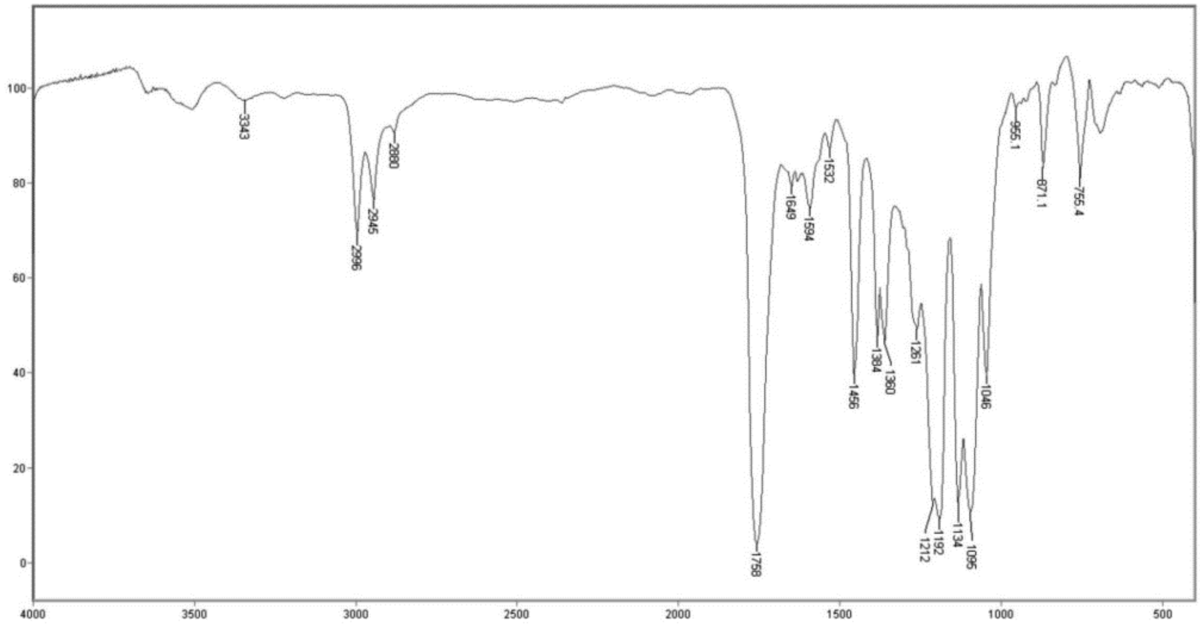

Figure S6. FTIR spectrum of prazosin-PLLA/Citr conjugate.

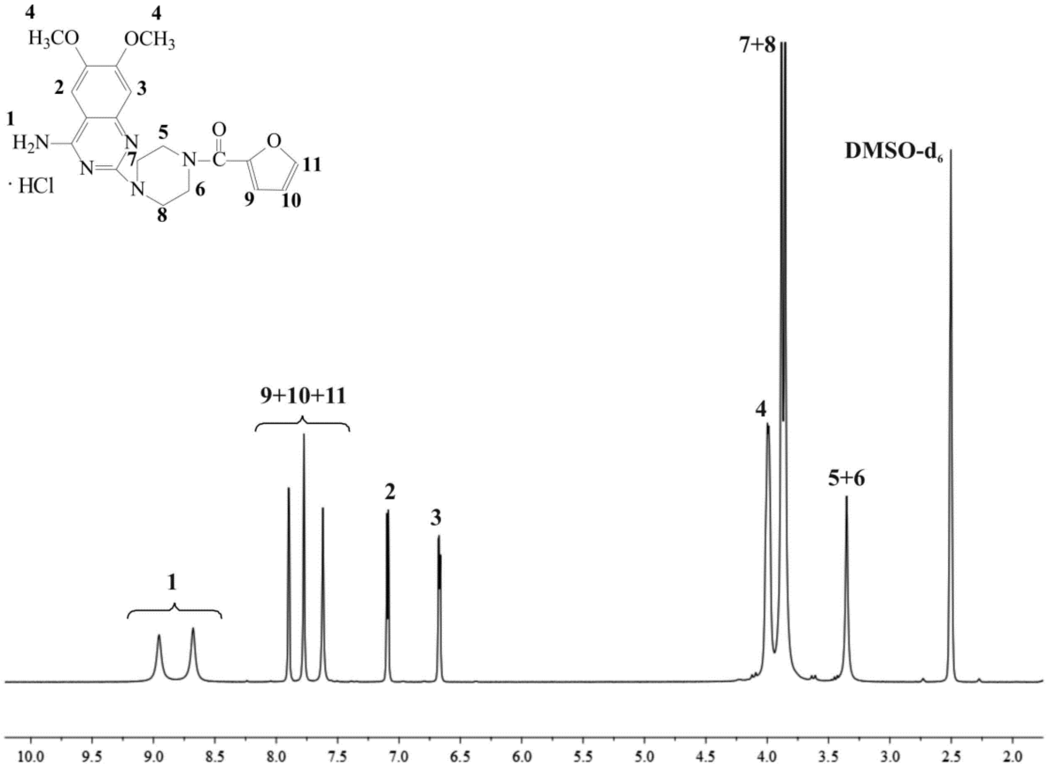

Figure S7. ^{1}H NMR spectrum of the prazosin hydrochloride.

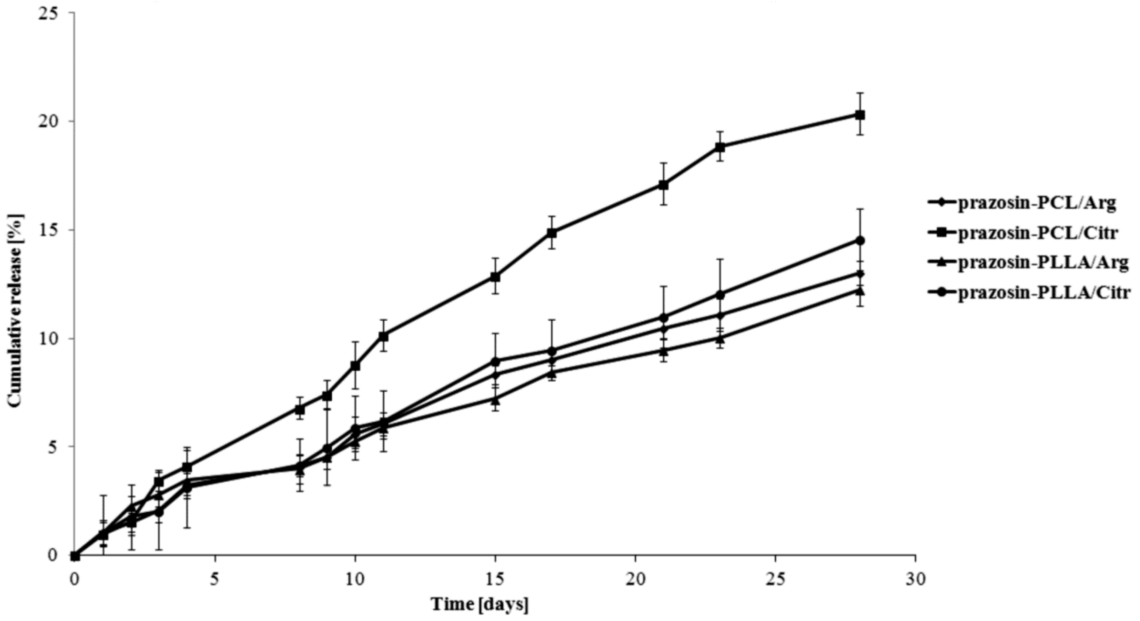

Figure S8. Release profile of prazosin from the synthesized conjugates (pH 7.4 ± 0.5).

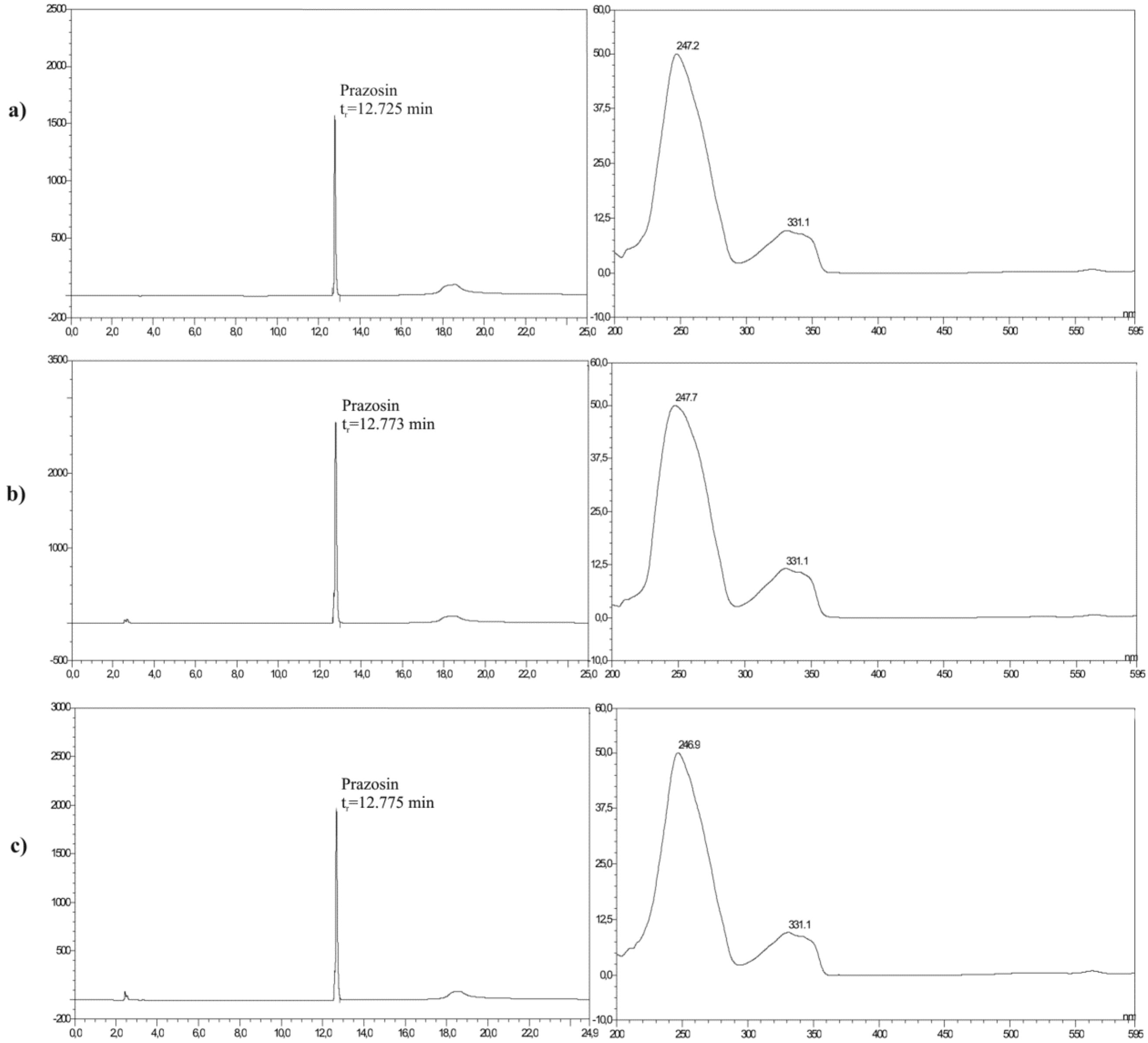

Figure S9. The HPLC chromatogram and UV spectrum of prazosin released from prazosin-PCL/Arg conjugate (before (**a**); after 9 (**b**) and 28 days (**c**) of degradation at pH 7.00 ± 0.05).
